# DiADEM—Dance against Dementia—Effect of a Six-Month Dance Intervention on Physical Fitness in Older Adults with Mild Cognitive Impairment: A Randomized, Controlled Trial

**DOI:** 10.3390/jpm14080888

**Published:** 2024-08-22

**Authors:** Ulrich Thiel, Marvin Stiebler, Berit K. Labott, Johanna Bappert, Corinna Langhans, Nicole Halfpaap, Bernhard Grässler, Fabian Herold, Stefanie Schreiber, Rüdiger Braun-Dullaeus, Patrick Müller, Notger Müller, Anita Hökelmann

**Affiliations:** 1 Department of Sport Science, Faculty of Humanities, Otto-von-Guericke University Magdeburg, 39104 Magdeburg, Germany; 2 Division of Cardiology and Angiology, University Hospital Magdeburg, 39120 Magdeburg, Germany; 3 Department of Intervention Research in Exercise Training, Institute of Exercise Training and Sport Informatics, German Sport University Cologne, 50933 Cologne, Germany; 4 Department of Neuromotor Behavior and Exercise, University of Muenster, 48149 Muenster, Germany; 5 Department of Neurology, Otto-von-Guericke University Magdeburg, 39120 Magdeburg, Germany; 6 Department of Degenerative and Chronic Diseases and Movement, Joint Faculty of Health Sciences, University of Potsdam, Brandenburg Medical School Theodor Fontane and Brandenburg Technical University Cottbus-Senftenberg, 14476 Potsdam, Germany; 7 Centre for Intervention and Research on Adaptive and Maladaptive Brain Circuits Underlying Mental Health (C-I-R-C), 39120 Magdeburg, Germany; 8 German Centre for Mental Health (DZPG), 39120 Magdeburg, Germany; 9 German Centre for Neurodegenerative Diseases (DZNE), 39120 Magdeburg, Germany; 10 Centre for Behavioural Brain Sciences (CBBS), 39120 Magdeburg, Germany

**Keywords:** geriatrics, prevention, sportive dance training, cardiorespiratory fitness, physical fitness

## Abstract

***Background***: Preserving health and physical fitness is critical to ensure independent living across the lifespan. Lower levels of physical fitness are associated with age-related cognitive decline and a higher prevalence of mild cognitive impairment (MCI). Thus, this study investigates the influence of a six-month dance intervention on selected measures of physical fitness in older adults with MCI. ***Methods***: In this randomized controlled trial, 55 patients with MCI were randomized into a sportive dance training (IG; *n* = 26; age: 70.7 ± 5.6 years; 62% female) or an inactive control group (CG; *n* = 24; age: 69.1 ± 6.8 years; 46% female). The dance group received two 90 min dance training sessions per week over a duration of six-months, which focused on learning dance movement patterns. During the training sessions, heart rate was measured to control exercise intensity. Physical fitness was assessed using cardiopulmonary exercise testing (CPET), lower limb functional fitness via sit-to-stand test, handgrip strength, and heart rate variability (HRV). ***Results***: We observed that the dance intervention preserved the cardiorespiratory fitness as measured by maximal oxygen uptake (VO_2max_) during CPET, which decreased in the CG. Furthermore, participants in the IG demonstrated increases in leg and handgrip strength, although these were not statistically significant. HRV displayed a non-significant decrease following the intervention. ***Conclusions***: The results of this randomized controlled trial suggest that sportive dance training can preserve elements of physical fitness (i.e., cardiorespiratory fitness) in older adults with MCI. Although improvements in the other parameters (i.e., leg and handgrip strength) were statistically non-significant, likely due to the small sample size, stabilizing muscular fitness and preventing age-related decline in older adults with MCI is important for maintaining functional independence. For future studies, we recommend a longer training duration paired with precise control of regular physical activity levels, an important confounding factor.

## 1. Introduction

Aging is inherently variable, with progressive physiological, motor, and cognitive decline accompanying the process. The global demographic shift, especially in industrial societies, underscores the importance of maintaining health and physical capacity in older adults. As the incidence and prevalence of chronic non-communicable diseases (NCDs) correlate with age, strategies aimed at extending the individual health span are receiving growing attention to preserve quality of life and reduce socioeconomic impact [1].

Central to this challenge is the maintenance of physical and cognitive fitness, which play a pivotal role in preserving autonomy and enhancing overall well-being in older adults [2]. Currently, the World Health Organization recommends a minimum of 150–300 min of moderate-intensity or 75–150 min of vigorous-intensity aerobic physical activity (PA) per week, along with targeted muscle strengthening twice a week [3] for all adults.

Key markers of physical fitness include, but are not limited to, cardiorespiratory fitness (e.g., as measured by maximal oxygen uptake [VO_2max_]), overall strength (e.g., handgrip strength), lower limb muscular strength (e.g., assessed via the sit-to-stand test), and the activity of the autonomous nervous system (e.g., heart rate variability [HRV]). These parameters can add a valuable dimension to our understanding of health management in an aging society and can help to conduct health-related decision making based on objective measures.

Overall, the evidence highlights a link between PA and the risk for age-related diseases [4]. Especially, physical exercise, as a subcategory of physical activity, is deemed a structured and repetitive effort in order to improve or maintain physical fitness [5]. Even when started at old age, physical exercise improves physical capacity, markers of cardiometabolic health and neuroplasticity, and reduces the risk of all-cause mortality [6]. Conversely, age-related decreases in physical fitness are associated with an increased risk of dementia, cardiovascular disease, and other NCDs [7,8,9].

Although the factors that contribute to healthy aging, including the prevention of disease, are numerous, being more physically active can increase physical fitness, an important predictor of health, regardless of age. This is the result of adaptations in various physiological systems, most notably within the neuromuscular system for better movement coordination, the cardiopulmonary system for more efficient oxygen and nutrient distribution throughout the body, and metabolic processes, particularly those that regulate glucose and fatty acid metabolism. Together, these adaptations can enhance overall physical fitness, which can ultimately converge in the better health of older adults [10,11].

Structured exercise programs present a variety of direct health benefits, for example, in mitigating sarcopenia and delaying age-related functional disability [12]. Adopting a lifestyle that meets the weekly recommendations of 150 min of moderate-intensity aerobic activity lowers the risk of cardiovascular diseases, their associated mortalities, and the incidence of type 2 diabetes [13,14]. In addition, evidence from epidemiological cross-sectional and intervention studies also suggests an association between PA and a reduced risk of dementia and mortality [15].

However, it remains unclear which type of physical exercise (e.g., aerobics, resistance, motor-coordinative) is most effective in preserving cognitive and physical fitness [16]. Overall, studies from animal models indicate that PA combined with an enriched environment including social contact can be most effective for inducing neuroplasticity [17].

A potential equivalent to the combination of PA and social enrichment is dancing. Dance, by nature, requires coordination, rhythm, and cognitive engagement and thus offers a multifaceted approach to exercise that may yield significant benefits beyond those of traditional PA (e.g., aerobic PA). Dancing is characterized by acyclic and coordinatively demanding movements and a variety of cognitive processes, including the learning, storage, and retrieval of new movement patterns. This involves and trains motor, sensory, and cognitive skills. Additionally, dancing is a sport with close social contact with peers, promoting social interactions that can lead to the formation of strong neural networks [18]. In this context, it can be hypothesized that social interactions like caring for dance partners and interacting with them during dance choreographies enhance the social life of older adults [18,19]. These social interactions emerging during dance training are likely to evoke a stronger activation of specific neural networks like the mirror neuron system, leading to significant activations in different cortices, for example, the parietal or parahippocampal cortices, thereby aiding the preservation of cognitive performance in aging by social enrichment [20].

Based on previous work [21,22], multi-modal approaches, such as dance/movement training, aim to impact both cognition and physical functioning in older adults. By improving both cardiorespiratory (VO_2max_) and muscular fitness (muscle hypertrophy and functional ability) within a single exercise regimen, these studies have shown promising results in young and older populations [23,24].

In addition, findings indicate that multimodal dance training can reduce the risk of dementia and make an important contribution to neuroplasticity and fall prevention, and thus preserve autonomy in old age; for example, Verghese and colleagues [25] report, in an analysis of 469 healthy seniors aged over 75 years, a 76% reduction in the risk of dementia through dancing.

Among the myriad of challenges faced by older adults, cognitive decline stands out. Particularly, MCI, as a transitional stage between the expected cognitive decline of normal aging and dementia [26], may constitute an important window of opportunity to modify the trajectory of cognitive aging by lifestyle interventions. Age-related cognitive decline and MCI are estimated to affect one in six people worldwide by 2050 [27]. The conversion rate from MCI to dementia is estimated to be around 10–15% per year [28,29]. In MCI, unlike dementia, daily living abilities are still preserved [29]. The protective effects of PA on dementia and overall cognitive function have been well-documented, underscoring the importance of maintaining an active lifestyle to counteract the cognitive decline associated with aging [15,30]. For example, during one year of aerobic exercise training, Erickson et al. [31] were able to show that cardiorespiratory fitness increases the size of the hippocampus and improves memory in older adults. Furthermore, Colcombe et al. [32] have shown that cardiorespiratory fitness is associated with reduced brain tissue loss in aging humans.

However, although engaging in PA, especially physical exercise, can be an important factor in reducing the risk of all-cause dementia and improving various markers of physical fitness, the evidence of the influence of dance training on physical fitness parameters in older adults diagnosed with MCI remains sparse. To bridge this gap, we conducted a randomized controlled trial in older adults with MCI, examining the effects of a six-month dance intervention on selected parameters of physical fitness. By focusing on dancing, this study aims to shed light on the potential of sportive dance training to enhance selected parameters of physical fitness in older adults with MCI, which can help to inform effective strategies for managing an age-related decline in physical fitness among this population.

## 2. Materials and Methods

### 2.1. Participants

Older adults with mild cognitive impairment (MCI) were recruited as part of the “DiADEM-Dance Against DEMentia” research project through advertisements in local newspapers, flyers, posters, word of mouth, and by using existing databases. During recruitment, the individuals were screened for eligibility based on the following inclusion criteria: (i) 50 to 80 years old, (ii) native German-speaking, and (iii) able to manage everyday activities independently. Individuals who had poor or uncorrected vision/hearing or color weakness/blindness, and/or suffered from (a) severe psychiatric disorders (e.g., bipolar disorder) or depression (assessed via the Geriatric Depression Scale (GDS; 15 items; cut-off score ≥ 6), (b) severe orthopedic diseases (e.g., a bone fracture in last six months, herniated vertebral disk), (c) severe muscular diseases (e.g., myositis, tendovaginitis), (d) severe cardiovascular diseases (e.g., heart insufficiency), (e) severe endocrinologic diseases (e.g., manifest hypothyroidism or hyperthyroidism, insulin-dependent diabetes mellitus type II, BMI > 30), (f) neurological diseases other than MCI (e.g., stroke, epilepsy, multiple sclerosis), (g) major injury or had major surgery in the last six months, and/or used neuroleptics, narcotic analgesics, benzodiazepines, or psychoactive medications, and/or consumed illegal intoxicants and/or had an alcohol abuse, and (h) were pregnant were excluded.

Participants with MCI were identified by a trained neurologist in accordance with Petersen et al. [33]. Thereby, objective cognition deficits were detected using the CERAS-plus test battery (1.5 z-scores below the age- and education-adjusted reference sample in at least one subtest).

With an effect size of partial η^2^ = 0.14 and a power of 0.8, 52 participants were required to detect significant effects of interaction in a mixed ANOVA (group × time) with a significance level of α = 5% [34].

**Figure 1:**
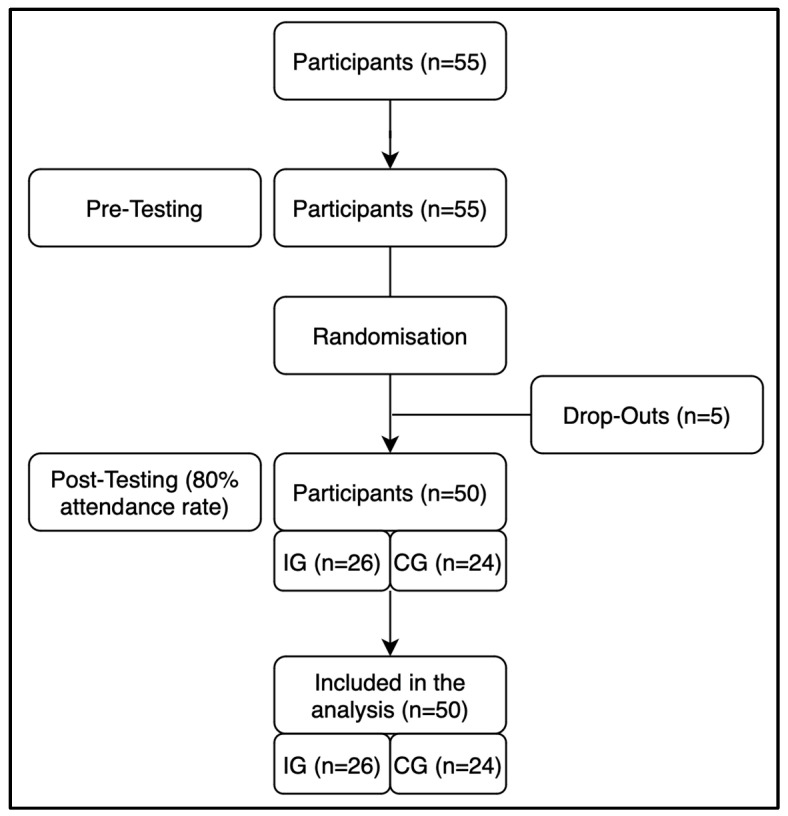
Flowchart of the study design.

All participants who were diagnosed with MCI were offered to take part in the intervention study. A total of 55 participants were split into two cohorts and then randomly allocated through stratified sampling (age and gender) into either an inactive control group (CG) or an intervention group (IG) participating in the dance training intervention. All participants were asked to maintain their usual lifestyle behavior, with the intervention group additionally participating in the administered dance training twice per week. One adult from each group swapped their originally assigned group to ensure that a married couple could participate in the same group. Five participants dropped out during the intervention period. All other participants demonstrated an attendance of at least 80% and were thus included in the post-measurement phase (Figure 1).

Prior to the assessment, participants were briefed about the experimental procedure and informed of possible risks and benefits associated with the study. All participants provided written consent to participate and received financial compensation. All study procedures were in accordance with the latest version of the Declaration of Helsinki and had been approved by the local ethics committee of the medical faculty of the Otto-von-Guericke University Magdeburg (reference number: 17/20). The intervention was registered in the German Clinical Trials Register (DRKS-ID: DRKS00022575) on the 5th of August 2020 and added to the Cochrane Central Register of Controlled Trials on 30th of November 2020 (CENTRAL-ID: CN-02186572).

### 2.2. Intervention

During the six-month intervention period, the sportive dance training group performed two 90 min training sessions per week. In order to control the training intensity (i.e., cardiovascular strain), the heart rate of each individual participant was measured with the Polar Team Pro System (Polar Electro GmbH, Büttelborn, Germany) during all training sessions. Thereto, the participants were asked to wear a heart rate sensor (Polar Pro Sensor, Polar Electro GmbH, Büttelborn, Germany), which was attached to their body with a chest strap just below the chest muscles. The average heart rate for each training session was recorded for all participants. At the beginning of each session, all participants sat quietly on the bench for 2 min to establish a baseline. The length of the chest strap was individually adjusted, and the participants wore the same sensor in each training session for the entire duration of the intervention. To individualize the sensors, the respective data (a) gender, (b) height, (c) age, (d) training volume per week, (e) maximal heart rate, (f) resting heart rate, (g) VO_2max_, (h) aerobic threshold, and (i) anaerobic threshold were entered in the system, of which parameters (e) to (i) were assessed through CPET.

A qualified instructor who held a master’s degree in sport science and was experienced in dance with older adults supervised each training session. The program of the sportive dance training focused on the continuous learning of new movement patterns and choreographies, including the training of coordination, and learning of specific dance skills. The participants performed the dance movements in order to memorize the movement combinations prior to dancing the step sequences. In addition, the orientation in space and the intensity were important in the execution of the dance routines, specifically the speed of movement, which was controlled by the instructors and progressively increased by adjusting the speed of the music. A multitude of individual dance genres was utilized, including line dance, jazz dance, square dance, and Latin-American dances. The training session was divided into four parts. The beginning was a warm-up with mobilization exercises for the whole body and the implementation of a coordinative part using various arm–leg combinations with increasing complexity. In addition, the dances that had already been rehearsed were gradually integrated into the warm-up during the program to activate the cardiovascular system and set a stimulus that triggers adaptations of the cardiovascular system. The main part was divided into two sections. The first section of the main part was strongly characterized by dance combinations that focused on complex coordinative tasks. Some of the choreographies in this part were danced with small equipment such as sticks, towels, or physio balls as drums. The second section of the main part focused on the strength-endurance aspects of dancing. Choreographies were employed and small fitness equipment such as brasils, fitness tires (1.2 kg), and small dumbbells were used for dancing to emphasize the physical demands of dancing. The last part of each training was a cool-down phase including a feet-to-head stretching routine and a sequence of low-intensity mobility exercises that was maintained over the entirety of the intervention. The participants could therefore internalize the routine sequences and execution of the exercises.

The training sessions aimed to constantly challenge the participants in a controlled manner with moderate intensity, as determined by the selected music. The exercise intensity was controlled by beats per minute of the selected music. The majority (80%) of the training was accompanied by music with an appropriate tempo, while the cool-down phase was typically performed with relaxing music. Different genres of music and rhythms were offered so that the training remained varied and different musical preferences catered to. Challenging dance steps were broken down into progressions and put together piece by piece to form the entire choreography and dance sequence. When most of the participants had clearly internalized all dance steps of the sequence, the tempo was, when possible, progressively increased until the intended, original tempo was reached.

### 2.3. Cardiopulmonary Exercise Testing

All participants completed an incremental step test to voluntary exhaustion to determine their individual VO_2max_ on a bicycle ergometer. This test is safe to perform in older adults [35] and has been shown to have high reliability [35,36]. The seat height was adjusted to attain a bend of 5° in the knee joint at the lowest position in the pedal revolution. Warming up included three-minute unloaded pedaling at 0 W. The resistance increased by 25 W every three minutes. Participants were required to keep the cadence between 60 and 70 revolutions per minute.

During the incremental cycling test, breath-by-breath pulmonary gas-exchange data (MetaSoft, Studio: Cortex Biophysik GmbH Leipzig, Germany), heart rate (Custo med 100, custo med GmbH, Ottobrunn, Germany), and lactate levels (Lactate Scout 4, EKF Diagnostic, Barleben, Germany) were assessed. The VO_2max_ and power output were normalized for body weight to account for anthropometric differences in the group of subjects using the following formulas: VO2 (mL/min)/kg and P (W)/kg. Ratings of perceived exertion were collected after each step using a 6–20 Borg Scale [37]. The highest stated value on the Borg Scale was used to measure the maximum perceived exertion.

The cardiopulmonary exercise testing (CPET) concluded when (i) the respiratory exchange ratio was above 1.10, (ii) a plateau in VO2 occurred (despite increasing workload), or (iii) the rating of perceived exertion was 18 or higher on the Borg Scale. CPET was terminated prematurely in the case of major electrocardiographic abnormalities, excessive blood pressure increase (≥230 mmHg systolic and/or ≥110 mmHg diastolic), or individual request [38].

### 2.4. Heart Rate Variability

The measurement of HRV was performed as follows: Subjects were seated comfortably in a chair with their knees bent at a 90° angle, and their hands resting on their thighs. They were instructed to relax and breathe normally throughout the procedure. To minimize artifacts, participants were instructed not to speak or move during the recording. A stabilization period of five minutes was implemented to ensure a relaxed state. Subsequently, the resting state measurement was recorded for another five minutes [39].

Electrocardiographic data were captured using a three-channel Holter-Electroencephalogram device, with a sampling rate of 1000 Hz (Medilog AR12plus, Schiller Medizintechnik GmbH, Baar, Switzerland). The raw electrocardiographic data were then uploaded to the Medilog Darwin 2 analysis software package (Schiller Medizintechnik GmbH, Baar, Switzerland) for automatic analysis. A healthcare professional visually examined the data for any clinical abnormalities. Text files containing consecutive NN intervals were generated for further analysis.

The HRV analysis was performed using the Kubios premium 3.3 software package (University of Kuopio, Kuopio, Finland). Artifact correction was conducted with an artifact identification threshold of 0.3 s. The detrending of NN intervals utilized the smoothness priors method with parameters Lambda = 500 and fc = 0.035 Hz, following national guidelines and the relevant literature [40,41].

The HRV analysis encompassed time-domain, frequency-domain, and non-linear parameters. Mean heart rate (mHR) was measured in beats per minute. The time-domain index, RMSSD (root mean square of successive differences), as well as SDNN (standard deviation of all NN intervals) were measured in milliseconds. HF (high-frequency power) is given in ms^2^. As non-linear measures have been relatively underutilized in the assessment of cardiac autonomic control, the non-linear index D2 was employed as a parameter reflecting the heart rate complexity. Higher D2 values indicate a greater complexity and adaptability of the cardiac system, while lower values suggest a shift toward sympathetic dominance [42].

### 2.5. Handgrip Strength

The assessment of maximal handgrip strength was conducted following the Southampton protocol [43]. Measurements of handgrip strength obtained by dynamometry show good-to-excellent relative reliability, but more variability is seen in absolute reliability, hence relatively large percentage changes would be necessary to indicate significant changes over time [44]. To measure maximal handgrip strength, participants were asked (i) to sit in a chair with their feet flat on the ground, (ii) to adduct their shoulders and maintain their arm in a position with neutral rotation, (iii) to flex their elbow at a 90-degree angle, while the wrist maintained a neutral position (i.e., thumb facing upward), (iv) to exert maximum force by squeezing their hand as hard as possible for a duration of three seconds.

For each hand, participants performed three trials, switching hands after completing one trial [45,46,47]. The trial that yielded the highest absolute value for handgrip strength (measured in kg) was used for further statistical analysis.

To account for the influence of body composition, the maximum handgrip strength was then normalized to the body weight of the subject using the following formula: normalized handgrip strength = absolute handgrip strength (in kg)/body weight (in kg) [48]. 

### 2.6. Sit-to-Stand Test

The functional strength of the lower body was quantified using the 30 s sit-to-stand test, which is a reliable and valid tool for the quantification of lower body strength in populations of active, older adults and is often incorporated into fall risk assessments [49]. During the 30 s sit-to-stand test, the participants were instructed to rise from a chair of standard height (43 cm) without armrests and sit down afterward. They were required to perform the task for thirty seconds, aiming to complete as many repetitions as possible while keeping their arms folded in front of their chest. The test was conducted without wearing shoes, and the performance was quantified in the number of completed repetitions during the 30 s, from the first initial seated position to the final seated position.

### 2.7. Statistical Analysis

Statistical analysis was performed using SPSS (IBM SPSS Statistics for Windows, Version 28.0. Armonk, NY, USA: IBM Corp). Normal distribution was tested using the Shapiro–Wilk test as well as being visually inspected in Q-Q plots. Outliers were detected via boxplot diagrams, checked for likelihood of occurrence by deviation and dismissed when deemed a measurement error. Descriptive statistics (mean ± standard deviation [SD]) were calculated for all included parameters. For inferential statistics, repeated measures analysis of variance (ANOVA) with a 2 × 2 configuration with time (pre, post) as the within-subject factor and group (control, intervention) as the between-subject factor were performed. Levene’s test was used to assess the homogeneity of error variances. In case of heterogeneity, a power transform via box-cox-transformation was used to stabilize the data. Effect size was calculated as partial η^2^ and interpreted as follows: ≥0.01 to <0.6: small effect; ≥0.06 to <0.14: medium effect; ≥0.14: large effect [50]. For all tests, the significance level was set at α = 5%.

## 3. Results

Out of all recruits, 55 were eligible to participate in this study. Five participants dropped out during the intervention period because of a non-intervention-related occurrence of back pain (*n* = 1), diseases of the heart (*n* = 1), or lack of time/interest (*n* = 3). All participants showed attendance rates of above 80% during training, thus no participants were excluded due to low attendance. In all, a total of 50 participants were eligible for final analysis, with 26 participants in the intervention group and 24 participants in the control group (Table 1). For the analysis of physical fitness, four participants did not attend the testing of handgrip strength and sit-to-stand performance, leaving 46 complete datasets for the analysis. For heart rate variability, ten participants were dismissed for skewed results. Borg values of the CPET were excluded from the ANOVA since they did not show the homogeneity of mean error variances. No falls or other adverse events occurred during the training intervention.

### 3.1. Exercise Intensity

**Figure 2:**
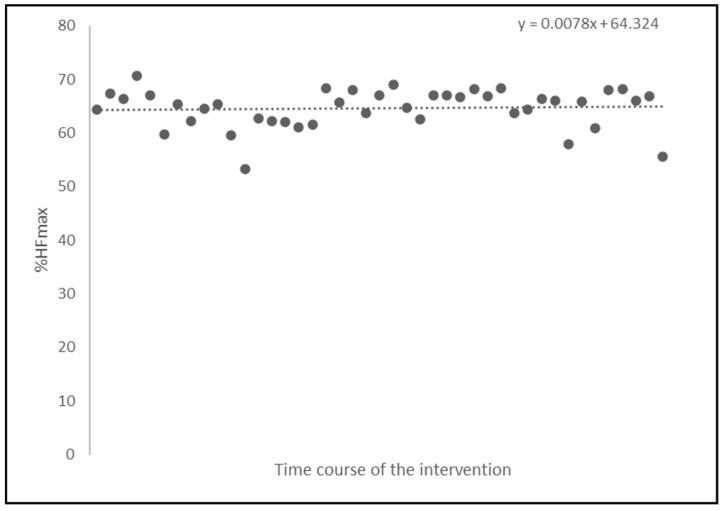
Development of average exercise intensity calculated as %HF_max_ following the time course of the intervention over 43 training sessions, with a dotted line illustrating the trendline.

Heart rate was monitored during each 90 min training session to gauge exercise intensity and guide the modification of the exercise intensity through the adjustments of the external load in the subsequent dance training sessions. Exercise intensity, i.e., internal load, was determined as the average percentage of the maximum heart frequency (%HF_max_ obtained during the CPET) across all training sessions for each participant of the intervention group. The average heart frequency measured throughout all training sessions for all participants was 97 ± 13.49 beats per minute. The average maximal heart rate for the participants was 149.3 ± 16.7 bpm (as determined by CPET). The average exercise intensity for the intervention group remained stable at %HF_max_ = 64.32% throughout the intervention (Figure 2).

### 3.2. Cardiopulmonary Exercise Testing

There was a statistically significant interaction between time and group for VO_2max_: F(1, 35) = 4.326; *p* = 0.045, partial η^2^ = 0.110 (Table 2). Regarding the control group, cardiorespiratory fitness, operationalized by VO_2max_, decreased from pre-test to post-test (*F*(1, 14) = 9.448, *p* = 0.008, partial η^2^ = 0.403), while such an effect of time was not noticed in the intervention group (F(1, 21) = 0.346, *p* = 0.563, partial η^2^ = 0.016).

There was no statistically significant interaction between time and group for any other parameter (P_max_: F(1, 35) = 1.013, *p* = 0.321, partial η^2^ = 0.028, HF_max_: F(1, 35) = 0.015, *p* = 0.903, partial η^2^ = 0.000, Lactate: F(1, 35) = 0.703, *p* = 0.408, partial η^2^ = 0.020).

There was a statistically significant main effect for time for P_max_: *F*(1, 35) = 5.250, *p* = 0.028, partial η^2^ = 0.130, but not for HF_max_: *F*(1, 35) = 1.682, *p* = 0.203, partial η^2^ = 0.046 or Lactate: *F*(1, 35) = 1.063, *p* = 0.310, partial η^2^ = 0.029.

There was no significant main effect for group for the parameters P_max_: *F*(1, 35) = 0.230, *p* = 0.634, partial η^2^ = 0.007; HF_max_: *F*(1, 35) = 0.929, *p* = 0.342, partial η^2^ = 0.026; and Lactate: *F*(1, 35) = 0.081, *p* = 0.777, partial η^2^ = 0.002.

### 3.3. Sit-to-Stand Test

There was no statistically significant interaction between time and group, *F*(1, 44) = 3.053, *p* = 0.088, partial η^2^ = 0.065 (Table 3). However, we noticed a significant main effect for time: *F*(1, 44) = 19.535, *p* < 0.001, partial η^2^ = 0.307, whereby both groups showed increased performance from pre-test to post-test. No significant main effect for group emerged: *F*(1, 44) = 18.47, *p* = 0.270, partial η^2^ = 0.28.

### 3.4. Heart Rate Variability

There was no statistically significant interaction between time and group for any parameter (mHR: *F*(1, 37) = 2.210, *p* = 0.146, partial η^2^ = 0.056, SDNN: *F*(1, 37) = 0.924, *p* = 0.566, partial η^2^ = 0.000, RMSSD: *F*(1, 37) = 0.004, *p* = 0.948, partial η^2^ = 0.000, HF: *F*(1, 37) = 0.698, *p* = 0.566, partial η^2^ = 0.004, D2: *F*(1, 37) = 0.222, *p* = 0.641, partial η^2^ = 0.006) (Table 4).

There was no significant main effect for time for any parameter (mHR: *F*(1, 37) = 2.625, *p* = 0.114, partial η^2^ = 0.066, SDNN: *F*(1, 37) = 1.799, *p* = 0.188, partial η^2^ = 0.046, RMSSD: *F*(1, 37) = 3.496, *p* = 0.069, partial η^2^ = 0.086, HF: *F*(1, 37) = 0.153, *p* = 0.698, partial η^2^ = 0.004, D2: *F*(1, 37) = 2.419, *p* = 0.128, partial η^2^ = 0.061).

There was no significant main effect for group for any parameter (mHR: *F*(1, 37) = 0.148, *p* = 0.703, partial η^2^ = 0.004, SDNN: *F*(1, 37) = 0.019, *p* = 0.891, partial η^2^ = 0.001, RMSSD: *F*(1, 37) = 0.001, *p* = 0.977, partial η^2^ = 0.000, HF: *F*(1, 37) = 0.153, *p* = 0.698, partial η^2^ = 0.004, D2: *F*(1, 37) = 0.152, *p* = 0.698, partial η^2^ = 0.004).

### 3.5. Handgrip Strength

There was no statistically significant interaction between time and group, *F*(1, 44) = 0.335, *p* = 0.566, partial η^2^ = 0.008 (Table 5). A significant main effect for time was observed with *F*(1, 44) = 4.409, *p* = 0.042, partial η^2^ = 0.091, considering that both groups showed increased handgrip strength from pre-test to post-test. Handgrip strength was typically higher for the right hand, as indicated by a significant main effect for hand: F(1, 44) = 7.917, *p* = 0.007; partial η^2^ = 0.152. There was no significant main effect for group, *F*(1, 44) = 0.708, *p* = 0.405, partial η^2^ = 0.016.

## 4. Discussion

This study examined the effects of a six-month sportive dance intervention on selected parameters of physical fitness in older adults with MCI. Our findings contribute to the growing body of evidence supporting the benefits of structured forms of PA, particularly dance, in mitigating age-related decline in physical fitness [1,21,51,52,53,54].

Overall, the intervention group demonstrated mostly improvements or stabilization in general fitness parameters compared to the control group.

### 4.1. Cardiorespiratory Fitness

Notably, participants in the intervention group showed no decline in VO_2max_ (i.e., preservation of cardiorespiratory fitness), when compared to the control group, as evidenced by CPET. This aligns with previous research indicating that an endurance-based dance training can preserve cardiorespiratory fitness [24,51,55,56]. However, no significant improvements were observed in HF_max_, P_max_, and lactate levels, which could be attributed to the moderate intensity of the dance training with an average of 64% HF_max_. The movements of the different dance choreographies needed to be initially executed at a slower speed to allow the participants their correct encoding, probably resulting in a lower exercise intensity. In the literature, Karlsen et al. [57] emphasized that, among other factors (e.g., training duration), exercise intensity is an important moderator of the intervention-related changes in cardiorespiratory fitness.

### 4.2. Sit-to-Stand Test

The functional strength of the lower body, assessed using the 30 s sit-to-stand test, showed higher, although non-significant, improvements in the intervention group, when compared to the control group. The previous literature on the sit-to-stand test in older adults has shown variability in intervention-related changes in performance levels, but our results tendentially align with observations of studies that have observed a better performance after sportive dance training interventions [58,59,60].

### 4.3. Handgrip Strength

Our dance training did not show a significant effect on handgrip strength. Little research exists on handgrip strength following a dance intervention; however, Woloszyn et al. [61] noted an improvement in handgrip strength for older wheelchair users after finishing twelve weeks of dance movement therapy, but it can be argued that the wheelchair use itself was a sufficient stimulus for increasing handgrip strength. Another study found a favorable effect for the control group after twelve weeks, while the dance group did not display significant changes [62]. A meta-analysis by Labott et al. [63] concluded that multimodal training approaches generally exert a stronger influence on handgrip strength than interventions using a single type of exercise (e.g., strength, balance, flexibility, or endurance). Given that in our study, both IG and CG improved from pre-testing to post-testing, the observed changes cannot be attributed to the intervention, although small training gadgets such as weights and drumsticks were regularly used throughout the dance training.

### 4.4. Heart Rate Variability

Comparable to handgrip strength, no statistically significant influence of the dance training on any of the parameters of heart rate variability was noted. The lack of significant changes in HRV is particularly noteworthy, as a higher HRV is generally associated with better cardiovascular health [64,65,66,67]. The decrease or preservation of the parameters of HRV in this study may reflect age-related limitations in the cardiovascular system’s adaptive capacity [68].

### 4.5. Strengths and Limitations

One strength of this study is the intervention period of six months, whereas some research only ensures a training duration of twelve weeks [51,60]. Additionally, real-time assessment of HR through continuous heart rate measurements has shown to ensure both adequate and stable exercise intensity of the training sessions over the intervention period.

A few limitations should be considered when interpreting these findings. During CPET, psychological factors and varying levels of motivation among participants may have impacted their performance during these tests.

Additionally, the roof of the sports room used for the dance training sessions interfered with the GPS sensors of the heart rate sensor, whereby the recorded speed of the participants as well as the traveled distance as markers of external load could not be evaluated.

Moreover, handgrip strength or squatting (sit-to-stand) were not specifically trained during the intervention. In addition, strength stimuli during the intervention might have been received differently by the opposite sexes, which could lead to greater variation in the performance results, i.e., heterogeneity and, hence, statistically non-significant findings.

The COVID-19 pandemic also influenced the study design and participant lifestyle behaviors. The need to adhere to pandemic regulations resulted in the formation of two cohorts, whereby the initially planned intervention duration was halved, from twelve to six months. Hypothetically, a longer duration would have led to more pronounced effects on our selected parameters of physical fitness. Additionally, the pandemic regulations could have potentially increased sedentary behavior in both groups due to homebound restrictions, possibly skewing the magnitude of effect and making the results more difficult to compare to studies in non-pandemic times. Thus, future research should aim to replicate this study under non-pandemic conditions.

In light of the expected demographic change, interventions aimed at prevention by improving lifestyle factors such as PA are important for older, as well as younger, populations. However, the feasibility and effectiveness of interventions in older people compared to younger populations are of major concern. Sedentary behavior and pre-existing conditions increase with age, and physical fitness declines, limiting study outcomes and increasing the risk of injury during intervention. Moreover, pre-existing conditions can exclude a large number of older adults from study participation, limiting the applicability of the findings to an even smaller subset of older adults and making generalizations impossible. Conversely, interventions in younger populations may be more feasible and more effective in the magnitude of the results, but findings cannot be generalized to an older population due to the aforementioned circumstances. Hence, more studies in an older population and subsets thereof, as this study aimed to do, are necessary, though great care should be taken in conducting any intervention.

## 5. Conclusions

In conclusion, our study supports the potential of dance interventions to preserve aspects of physical fitness in older adults with MCI, particularly cardiorespiratory fitness (operationalized by VO_2max_). However, the intervention did not significantly change other parameters of cardiorespiratory fitness (e.g., P_max_, HF_max_, and maximum lactate levels), functional lower body strength, handgrip strength, or HRV.

The promising results regarding the preservation of physical fitness in our cohort of older adults with MCI, especially of cardiorespiratory fitness, suggest that dance could be a valuable intervention to allow for a healthy aging of older adults. Given its multimodal nature, combining physical, cognitive, and social elements, dance offers a holistic approach to enhancing the health and well-being of this population. Further investigation into the specific psychophysiological mechanisms underlying these benefits will be crucial in optimizing dance-based interventions and maximizing their impact on healthy aging.

Overall, this study underscores the potential of dance as an effective and enjoyable form of structured physical activity for older adults.

## Figures and Tables

**Table 1:** Participant characteristics. Values are mean ± standard deviation.

Characteristics	Total	Intervention Group	Control Group	*p*-Value
**Total (*n*)**	50	26	24	
**Sex (n_female_/n_male_)**	27/23	16/10	11/13	0.275
**Age (years)**	69.9 ± 6.2	70.7 ± 5.6	69.1 ± 6.8	0.373
**Height (cm)**	171.5 ± 9.2	169.1 ± 8.9	174 ± 9	0.060
**Weight (kg)**	74.4 ± 11.3	71.8 ± 8.2	77.1 ± 13.5	0.097
**Years of Education**	15.6 ± 2.5	15.8 ± 2.7	15.3 ± 2.1	0.396
**Score MMSE**	27.2 ± 1.4	27.1 ± 1.5	27.3 ± 1.2	0.651

**Table 2:** Mean and standard deviation of cardiorespiratory fitness in both groups.

Variables	CG		IG		ANOVA (Group × Time)	
	Pre (Mean ± SD)	Post (Mean ± SD)	Pre (Mean ± SD)	Post (Mean ± SD)	F	*p*
**P_max_**	133.33 ± 40.83	121.67 ± 42.11	123.86 ± 35.76	119.32 ± 36.13	1.013	0.321
**VO_2max_ (mL/min/kg)**	26.47 ± 6.51	24.4 ± 5.97	25.45 ± 5.99	26.00 ± 6.98	4.326	0.045 *
**HF_max_ (bpm)**	152.27 ± 17.29	149.33 ± 17.17	147.68 ± 15.27	144.14 ± 17.96	0.015	0.903
**Max. lactate (mmol/L)**	6.26 ± 2.36	6.20 ± 3.21	6.30 ± 2.30	5.72 ± 2.04	0.703	0.408
**Borg**	17.00 ± 1.81	17.60 ± 1.40	16.95 ± 1.46	16.32 ± 2.08	n.c.	

bpm = beats per minute, n.c. = not calculated because of heterogeneity of mean error variances, *: level of significance *p* < 0.05.

**Table 3:** Mean and standard deviation of number of repetitions during sit-to-stand test in both groups.

Variable	CG		IG		ANOVA (Group × Time)	
	Pre (Mean ± SD)	Post (Mean ± SD)	Pre (Mean ± SD)	Post (Mean ± SD)	F	*p*
**Repetitions**	12.30 ± 3.03	13.20 ± 2.53	12.62 ± 2.70	14.69 ± 3.39	3.053	0.088

Level of significance *p* < 0.05.

**Table 4:** Mean and standard deviation of heart rate variability in both groups.

Variables	CG		IG		ANOVA (Group × Time)	
	Pre (Mean ± SD)	Post (Mean ± SD)	Pre (Mean ± SD)	Post (Mean ± SD)	F	*p*
**mHR (bpm)**	68.31 ± 11.32	64.83 ± 8.48	65.51 ± 9.30	65.36 ± 9.74	2.210	0.146
**SDNN (ms)**	25.51 ± 14.58	22.93 ± 9.56	24.80 ± 15.75	22.57 ± 11.69	0.924	0.566
**RMSSD (ms)**	27.22 ± 16.93	23.02 ± 12.24	27.51 ± 20.73	23.00 ± 14.80	0.004	0.948
**HF (ms^2^)**	251.35 ± 310.47	227.89 ± 236.24	336.22 ± 467.33	263.51 ± 312.19	0.698	0.566
**D2 (A.u.)**	0.71 ± 1.21	0.52 ± 0.85	0.93 ± 1.48	0.57 ± 1.05	0.222	0.641

mHR = mean heart rate, SDNN = standard deviation of all NN intervals, RMSSD = root mean square of successive differences, HF = high frequency power, BPM = beats per minute, A.u. = arbitrary units, level of significance *p* < 0.05.

**Table 5:** Mean and standard deviation of handgrip strength in both groups.

Variables	CG		IG		ANOVA (Group × Time)	
	Pre (Mean ± SD)	Post (Mean ± SD)	Pre (Mean ± SD)	Post (Mean ± SD)	F	*p*
**Handgrip Strength—Left Hand (normalized, A.u.)**	1.35 ± 0.46	1.41 ± 0.42	1.26 ± 0.39	1.29 ± 0.39	0.335	0.566
**Handgrip Strength—Right Hand (normalized, A.u.)**	1.40 ± 0.45	1.46 ± 0.45	1.31 ± 0.40	1.35 ± 0.36		

A.u. = arbitrary units, level of significance *p* < 0.05.

## Data Availability

The raw data supporting the conclusions of this article will be made available by the authors on request.
